# Assessment of Cytocompatibility and Anti-Inflammatory (Inter)Actions of Genipin-Crosslinked Chitosan Powders

**DOI:** 10.3390/biology9070159

**Published:** 2020-07-08

**Authors:** Simona Dimida, Matteo Santin, Tiziano Verri, Amilcare Barca, Christian Demitri

**Affiliations:** 1 Biomaterial Laboratory, Department of Innovation for Engineering, University of Salento c/o Ecotekne, 73100 Lecce, Italy; simona.dimida@unisalento.it; 2 Centre for Regenerative Medicine and Devices, School of Pharmacy and Biomolecular Sciences, University of Brighton, Brighton BN24GJ, UK; m.santin@brighton.ac.uk; 3 Applied Physiology Laboratory, Department of Biological and Environmental Sciences and Technologies, University of Salento c/o Ecotekne, 73100 Lecce, Italy; tiziano.verri@unisalento.it

**Keywords:** chitosan, genipin, biomaterials, inflammation, cell–material interactions

## Abstract

Chitosan is a polysaccharide commonly used, together with its derivatives, in the preparation of hydrogel formulations, scaffolds and films for tissue engineering applications. Chitosan can be used as such, but it is commonly stabilized by means of chemical crosslinkers. Genipin is one of the crosslinkers that has been considered that is a crystalline powder extracted from the fruit of *Gardenia jasminoides* and processed to obtain an aglycon compound. Genipin is gaining interest in biological applications because of its natural origin and anti-inflammatory actions. In this paper, the ability of chitosan-based materials crosslinked with genipin to exert anti-inflammation properties in applications such as bone regeneration was studied. Powders obtained from chitosan–genipin scaffolds have been tested in order to mimic the natural degradation processes occurring during biomaterials implantation in vivo. The results from osteoblast-like cells showed that specific combinations of chitosan and genipin stimulate high permissiveness towards cells, with higher performance than the pure chitosan. In parallel, evidences from monocyte-like cells showed that the crosslinker, genipin, seems to promote slowing of the monocyte-macrophage transition at morphological level. This suggests a sort of modularity of pro-inflammatory versus anti-inflammatory behavior of our chitosan-based biomaterials. Being both the cell types exposed to microscale powders, as an added value our results bring information on the cell–material interactions in the degradative dynamics of chitosan scaffold structures during the physiological resorption processes.

## 1. Introduction

Due to its abundance, versatility and biocompatibility, the polysaccharide chitosan (CS) is currently investigated for a wide range of tissue engineering and biomedical applications [1]. CS and its derivatives are largely adopted in the formulation of hydrogels, sponges and films developed for tissue engineering scaffolds [2,3,4,5]. Being able to favor attachment and proliferation of bone-forming cells and formation of mineralized bone matrix, CS is especially attractive as bone scaffold material [6,7].

The chemical and physical properties of this polymer have been studied for over 30 years, as well as its biological responses [8]. Despite the wealth of existing literature about its biomedical uses [9,10], data regarding the nature and strength of immune responses (immunoreactivity) induced by CS, e.g., administered through parenteral injection and/or implantation, are scarce and conflicting. In this respect, CS has been sometimes viewed as an inactive biomaterial, other times as able to induce specific inflammatory responses by molecular recognition. In literature, the immunoreactivity of CS is dependent on its deacetylation degree, molecular weight and the endotoxin content from the deacetylation process of chitin [11,12,13]. Although many studies describe that CS may differently elicit immune responses, e.g., based on the different commercial source. On the other hand recent and less recent works agree on the definition of general pro-inflammatory properties of CS [14,15].

In biomaterials research targeting to biomedical uses, CS is usually stabilized by chemical cross-linkers [16]. Among these is counted the genipin (International Union of Pure and Applied Chemistry-IUPAC name: methyl(1R,4aS,7aS)-1-hydroxy-7-(hydroxymethyl)-1,4a,5,7a-tetrahydrocyclopenta[c]pyran-4-carboxylate), an aglycon compound extracted from the fruit of *Gardenia jasminoides*. In the last few years, the scientific interest in genipin utilization is increasing, due to its natural occurrence, potential cytocompatibility [17,18,19] and the ability to elicit anti-inflammatory activity [20]. Our previous investigations have been preliminarily addressed to the chemical–physical and mechanical suitability of genipin crosslinked CS scaffolds, specifically intended for bone repair and regeneration. In such research, different genipin concentrations have been described as modular factors in terms of porosity and mechanical strength of the scaffolds (by inducing different structures, cross-linking degrees and degradation rates), even though it did not exert a significantly different impact on the proliferation dynamics of bone-like cell types in the CS-based scaffolds [21].

Aim in the present work is to further study a CS-based material with biocompatible characteristics suitable for bone regeneration. The inflammatory response after implantation of biomaterials is a key step for the activation of regeneration processes (in fact, mechanisms triggering the inflammatory reaction are intertwined with the general responses to implantation). Nevertheless, it is a Janus-faced factor when becoming chronic as it impairs the integration with the surrounding tissue through the excessive recruitment of monocytic cells to the injury site that leads to encapsulation of the biomaterial in a fibrotic capsule [22]. In this view, here genipin was evaluated because of its dual role of crosslinker and anti-inflammatory agent which helps in enhancing both the structural material and biocompatibility of CS as biomaterials for bone tissue implantation. The aim of this study was to use of CS-based materials grinded into powder starting from the initial membrane/scaffold form in a way that could mimic the occurrence of natural degradation processes starting once the biomaterial is implanted in vivo. [23,24]. Thus, the material grinding and its cell-based analyses have the following objectives: (a) to investigate the interaction of cells with irregular (shapeless) scaffold fragments and (b) to detect biological effects of the genipin molecules putatively released from the fragmented scaffold on cells responding to the genipin’s anti-inflammatory properties. As follow up of a work previously published [21], we carried out cytocompatibility studies using the human osteoblast-like MG63 cells, widely adopted for in vitro bone research [25,26,27]. For further biological evaluation, we used the human monocyte-like THP-1 cells, to evaluate their morphological responses towards the suspension–adhesion transition under treatments with Phorbol-12-myristate-13-acetate (PMA), i.e., proinflammatory compound commonly used to induce the monocyte activation [28].

## 2. Materials and Methods

### 2.1. Materials

Chitosan (CS) at medium molecular weight, 75% deacetylation degree and viscosity 200–800 cps (1 wt % in 1% acetic acid, 25 °C) was purchased from Sigma-Aldrich (prod. N. 448877; CAS 9012-76-4; origin: shrimp shells), as well as acetic acid and ethanol (purity 98%). This CS has been selected among those with low and high molecular weight due to the balance between solubility and capability to react with genipin, producing hydrogel with modular mechanical properties. Genipin (purity 98% (HPLC)) was purchased from Wako chemicals USA (Richmond, VA, USA) (prod. N. 078-03021). All the products were used without any further purification. Eagle’s minimum essential medium (E-MEM), Roswell Park Memorial Institute medium (RPMI-1640), fetal bovine serum (FBS), L-glutammine, penicillin/streptomycin antibiotic mix, Dulbecco’s phosphate buffer saline (D-PBS), paraformaldehyde (PFA) and 3-(4,5-dimethylthiazol-2-yl)-2,5-diphenyltetrazolium bromide (MTT) were purchased from Sigma-Aldrich. All reagents, medium supplements and plastic-ware were purchased as the cell-culture was tested. Phorbol-12-myristate-13-acetate (PMA; prod. n. P8139; CAS 0016561298), propanol and HCl were purchased form Sigma-Aldrich.

### 2.2. Preparation of Material Powders

Different dry material powders were prepared starting from scaffold membranes synthesized by freeze-drying according to the method of our previous work [21], i.e., containing 1.5% (*w*/*v*) of CS and genipin content variable from 1% to 3.5% and 7.5% (*w*/*w*) of the dry CS. CS powder free of genipin was also prepared by the same method. Briefly, after synthesis membranes were processed by immersion in liquid nitrogen and subsequent crushing in a porcelain mortar under sterile conditions. The obtained powders are reported as GNp (genipin 1% of CS), GN1p (genipin 3.5%) and GN2p (genipin 7.5%). For cell culture experiments, all powders were dispersed in sterile Petri dishes and sterilized by UV exposure (2 h) before adding to the cell culture medium; the GNp, GN1p, GN2p and CS powders were added to the treatment solutions at concentrations 1.5 mg/mL, 0.15 mg/mL and 0.015 mg/mL (serial dilutions) in the cell culture medium.

### 2.3. Morphological Analysis of Powders by Scanning Electron Microscopy (SEM)

The surface morphology of the GNp, GN1p, GN2p and CS powders were analyzed by scanning electron microscopy (ZEISS EVO 40, Carl Zeiss AG, Oberkochen, Germany) in low-vacuum modality and applying a voltage of 25 kV. Dried samples were placed on SEM sample holder, using double-sided adhesive tape and then observed without any modification. Images were acquired at 500× magnifications.

### 2.4. Cell Culture

In vitro cytocompatibility of powders and the qualitative analysis of cell–material interactions were performed adopting the human MG-63 osteoblast-like cell line (ATCC^®^ number CRL-1427^™^). MG-63 cells were maintained in sterile plastic flasks with E-MEM supplemented with 10% (*v*/*v*) fetal bovine serum (FBS), 2 mM L-glutamine, 100 U/mL penicillin and 100 ng/mL streptomycin, in a water-saturated atmosphere of 5% CO_2_ and 95% air (37 °C). For propagation, cells at 70–90% confluence were washed twice in D-PBS and detached with a 0.3% (*v*/*v*) trypsin solution, and then harvested by centrifugation; cell pellets were finally resuspended and transferred to new flasks. For the proinflammatory assays, the THP-1 human-derived monocyte-like cells (ATCC^®^ number TIB-202^™^) were grown in suspension in RPMI-1640 medium supplemented with 10% FBS (*v*/*v*), 2 mM L-glutamine and penicillin/streptomycin (100 U/mL; 100 ng/mL resp.). For propagation (every 3–4 days), cells at a growth density between 5 × 10^5^ and 1 × 10^6^ cells/mL were harvested by centrifugation, diluted in fresh culture medium and finally transferred to new flasks. All experiments were performed using cells between passages 3 and 10.

### 2.5. Evaluation of Cell Viability by MTT Assays

The powders and genipin were tested in time/dose dependence analyses performed using the MG-63 cells. Briefly, 1 × 10^5^ cells/mL were seeded in 24-well plates. After 24 h, the growth medium was replaced by medium containing 1.5 mg/mL, 0.15 mg/mL and 0.15 mg/mL of GN1p, GN2p and CS powders; in parallel, the cytotoxic effects of free genipin (gn) were analyzed adding gn at 0.75 µg/mL and 1.65 µg/mL (i.e., genipin content nominally present in 1.5 mg of the synthesized GN1p and GN2p materials, respectively) to the culture medium subsequently diluted at 1:10 and 1:100 (in this way, gn effects were tested in the range from 0.75 × 10^−2^ to 1.65 µg/mL). After treatments, MTT (3 [4,5, Dimethylthiazol 2 y1] 2,5 diphenyltetrazolium bromide) assays [29] were performed by adding MTT solution in D-PBS to the culture medium, at the final concentration of 50 μg/mL. After 4 h incubation at 37 °C, the medium has been removed and cells were washed 2–3 times with D-PBS until complete removal of the material powders. Finally, 500 μL of 2-Propanol/HCl 1N solution were added to each well to lyse cell membranes and dissolve the dark-blue formazan crystals produced by MTT intracellular metabolization in viable cells. The optical densities (ODs) of solutions from each sample were measured at λ = 550 nm by spectrophotometry, with a Multiskan Fc Microplate Photometer (Thermo Fisher Scientific, Waltham, Massachusetts, USA). For each experimental condition, data derived from mean values of *n* = 6 biological replicates; all tests were performed trice. OD data were normalized and expressed as percentage of metabolic activity (viability) with respect to the control mean values (untreated cells, 100% viability).

### 2.6. Hematoxylin/Eosin Staining of MG3 Cells

MG63 cells were seeded on UV-sterilized coverslips laid at the bottom of 6-well plates (1.5 × 10^5^ cells per well); after 24 h (50% ≤ confluence ≤ 70%), cells were treated with the GNp, GN1p, GN2p and CS powders. After treatments, the medium was removed and coverslips were washed twice with D-PBS, afterward cells on coverslips were fixed by overnight incubation at 4 °C with 4% (*w*/*v*) paraformaldehyde (PFA) in D-PBS. The day after, a standard hematoxylin/eosin (HE) staining protocol was performed as follows: (1) D-PBS washing (3×, 10 min each); (2) washing (1×) with distilled water for 5 min; (3) hematoxylin staining for 15 min and quick washing with distilled water (1×); washing with running water for 15 min; (4) rapid washing with distilled water and staining by eosin (acidified with glacial acetic acid) for 1 min; (5) after washing with distilled water, dehydration by increasing concentrations of ethanol in alcohol/distilled water solutions (50%, 70%, 95% and 100% *v*/*v*, 2 min each); (6) rapid immersion in xylene (2 min) and (7) mounting on microscope slides with the Eukitt^®^ (Merck KGaA, Darmstadt, Germany) acrylic resin mix and 12–18 h drying before final image acquisition. Bright/fluorescence images of samples were acquired with a Nikon AZ-100 stereoscopy system equipped with the Nikon (Miniato, Japan) NIS-Elements D package software.

### 2.7. PMA (Phorbol 12-Myristate 13-Acetate) Treatments of THP-1 Cells

THP1 cells were seeded into gelatin-coated 6-well plates (7.5 × 10^4^ cells/well). The GNp, GN1p, GN2p, CS or the gn solutions were added (genipin concentrations were applied as described above) and cells were incubated for 24 h. Afterwards, cells were treated for 48 h with 100 nM PMA (Phorbol 12-Myristate 13-Acetate) that was added to the RPMI-1640 medium. THP-1 cells treated with PMA alone were used as a positive control. At the end of incubation, supernatants (containing both materials and non-adherent cells) were removed and adherent cells were washed with D-PBS. Cells were fixed on the well bottom by 4% PFA for 2 h at 25 °C and subsequently washed twice in D-PBS before image acquisition. For each treatment condition, two replicates were prepared. Four cell counts in four different areas of the two biological replicates were performed (cell counts *n* = 8). The morphological analysis of the suspension–adhesion transition of cells undergoing treatments has been performed by definition of three arbitrary stages corresponding to three morphological shapes: (a) MPM (monocytes, pre-macrophage): adhered monocyte-like cells; (b) AI (adhered stage I, low morphological complexity): adhered spindle-shaped cells with *n* = 1 pseudopodial elongation and (c) AII (adhered stage II): adhered cells with *n* ≥ 2 pseudopodial elongations (advanced morphological complexity).

### 2.8. Statistical Analysis

If not otherwise stated, statistical analysis of the differences in the measured values of the experiments was performed with a Student’s *t*-test. All data are presented as means ± standard error of mean (S.E.M.). Differences were considered statistically significant when the *p* values were *p* < 0.05.

## 3. Results and Discussions

### 3.1. Morphological Analysis of Material Powders by Using SEM

**Figure 1:**
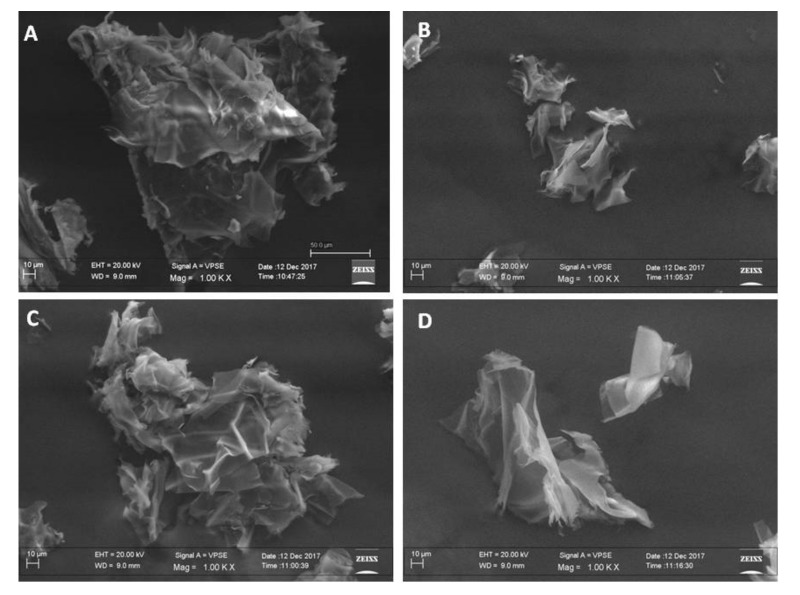
SEM morphological surface analysis of fragments from CS (**A**), GNp (**B**), GN1p (**C**) and GN2p (**D**) powders, 500× magnification.

Figure 1 shows SEM images of GNp (B), GN1p (C), GN2p (D) and CS (A) fragments from material powders. SEM images demonstrated that the freeze-drying method allows obtaining sample surfaces with high roughness, and interconnected porosity [21]. As expected, no morphological differences between materials powders containing different concentrations of genipin were highlighted, proving that genipin acts only at the molecular level. Conversely, the sample of pure CS seems to present some differences, especially if observed on the surface level, showing an increased thinner roughness, with less sharp edges. This can be due to the effect of the crosslink that, creating a stiffer polymer matrix, influences the growing rate of ice crystals in samples including genipin, during the freeze-drying treatment.

### 3.2. Evaluation of Cell Viability in the Presence of GNps, CS Powder and Genipin

As it is largely debated in literature about the dubious neutrality of CS in terms of immunoreactivity [11,12], likewise the effects of genipin in terms of cytocompatibility have not been comprehensively elucidated. As reported by several authors, genipin is less cytotoxic than chemical cross-linkers commonly used for modifying CS [17,18,19]. On the other hand, as a plant extract compound, it may produce adverse effects when used for in vivo biomedical applications [30]. In addition, genipin cytotoxicity can vary depending on the cell types, when tested in vitro [17], impeding a precise prediction of a safe range of concentrations to use for targeted applications. Based on our previous findings on the viability of MG63 osteoblast-like cells seeded and grown in genipin-crosslinked CS scaffold structures [21], the possible cytotoxic effects of materials reduced in powders, i.e., GN1p and GN2p, as well as of CS powder and gn were investigated. As the osteoblast-like cell model, MG63 are anchorage-dependent cells (ADCs), which are influenced by the extracellular matrix and the extracellular surface in growing and proliferating [31,32,33]. Overall, the in vitro expansion, growth and phenotype regulation of ADCs are processes directly depending on the mechanical and morphological features of the growth surface, which interacts with cell membranes at both the nano- and meso- or microscale level [34].

**Figure 2:**
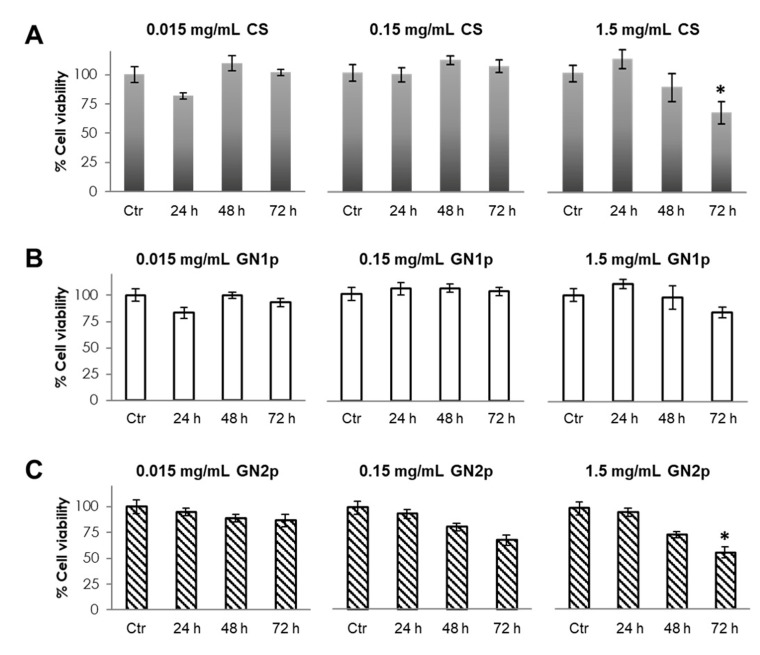
Analysis of the viability of MG63 cells in the presence of the CS, GN1p and GN2p powders. MTT assays were performed on MG63 cells cultured in the presence of serial dilutions (1.5, 0.15 and 0.015 mg/mL) of (**A**) CS powder, (**B**) GN1p and (**C**) GN2p at different time points (24, 48 and 72 h). Data are reported as mean values from *n* = 6 biological replicates and are expressed as % viability (± S.E.M.) with respect to the untreated control cells (Ctr) at each time point. Statistical analysis was assessed by a Student’s *t*-test (* *p* < 0.05).

Cell viability was investigated at 24, 48 and 72 h after exposure to the material powders and gn at different concentrations. As reported in Figure 2, after 72 h of exposure to the maximum tested concentration, i.e., 1.5 mg/mL, MG63 cells treated by the CS powder showed a time-dependent significant reduction of metabolic activity vs. the untreated control (66 vs. 100%, respectively), while no significant changes were detected at the lower concentrations (0.15 mg/mL and 0.015 mg/mL) at each time point (Figure 2A). When effects of the GN1p powder were analyzed, no significant reduction of cells metabolic activity was detected in treated cells vs. control, for each experimental concentration and/or time point (Figure 2B). On the other hand, by exposing cells to the presence of GN2p, a time-dependent decreasing trend was observed with both 0.15 mg/mL and 1.5 mg/mL concentrations, but metabolic activity was significantly lower with respect to untreated control only in the presence of the highest concentration at 72 h (59 vs. 100% respectively; Figure 2C).

It can be noticed that in this test, specifically, the GNp sample was not tested. For this formulation, the reaction kinetics were very low at room temperature. In fact, in a previous work we have demonstrated that the crosslinking reaction of GN1p and GN2p takes place only when the activation energy is greater than the thermal degradation [21]. This mechanism is a barrier to the production of reproducible scaffolds when the gn concentration is lower than the amount used for GN1p production, since to activate the reaction temperature should be used, and this promotes a consistent uncontrolled degradation of the polymer, thus implying lower structural interest.

**Figure 3:**
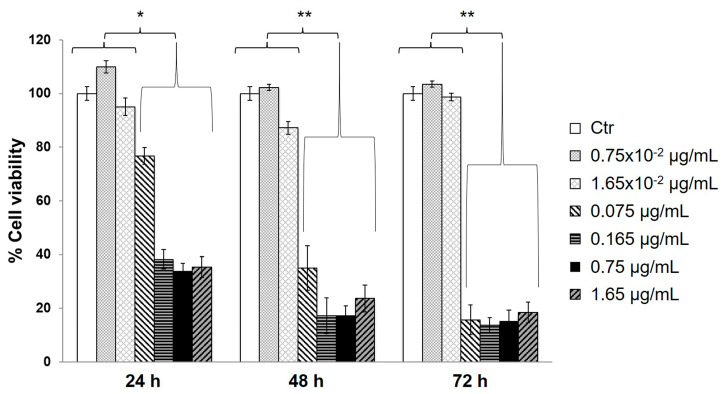
Analysis of viability of MG63 cells in the presence of serial dilutions of free genipin (gn). MTT assays were performed on MG63 cells cultured in the presence of gn solutions at different time points (24, 48 and 72 h). Data are reported as mean values from *n* = 6 biological replicates and are expressed as % viability (± S.E.M.) with respect to the untreated control cells (Ctr) at each time point. Statistical analysis: Student’s *t*-test (* *p* < 0.05; ** *p* < 0.01).

In parallel, the cytotoxic effects of gn were analyzed, from 2.2 × 10^−3^ to 1.65 µg/mL, chosen as a concentration range covering the genipin content according to the synthesis of GNp, GN1p and GN2p materials [21] and their dilutions as experimentally tested. As shown in Figure 3, with concentrations equal to or higher than 0.075 µg/mL, cell viability invariably decreased with the time-dependent trend from 24 to 72 h treatments. Additionally, it could be observed that no significant dose-dependent trend was detected with concentrations equal to or higher than 0.165 µg/mL, at each time point analyzed. By using the more diluted gn solutions (1.65 × 10^−2^ and 0.75 × 10^−2^ µg/mL), the overall viability changes were not significant with respect to the untreated control cells.

Taken together, the results gave interesting information about the cytocompatibility/toxicity of genipin as the constitutive component of the investigated material powders. In fact, it is noteworthy that at each time point, GN1p did not retain the toxicity shown by the corresponding amount of soluble gn, specifically at higher concentrations (see 0.075 and 0.75 µg/mL in Figure 3). Additionally, at 72 h and 1.5 mg/mL, cells grown in the presence of GN1p had a better performance in terms of metabolic activity (85% vs. the 100% of the untreated control, without statistical significance) than in the presence of the CS component alone (66% vs. 100% of the untreated control, statistically different). Interestingly, these findings could be ascribed to changes in the morphological and/or structural features of the different material powders [35]. This behavior can be explained with the differences in terms of the mechanical responses to the cell–material surface interaction between crosslinked (GN1p) and not crosslinked (CS) materials; genipin induces differences in the stiffness of the material by acting on the presence of crosslinks between the different chitosan backbones [21]. As the overall results suggest, it can be also noticed that GN2p induces a statistically significant reduction of the metabolic activity of cells with respect to its control whilst metabolic activity is not statistically different from the control in the presence of GN1p; despite this, GN2p does not show the strong toxicity of its own genipin content administered at higher concentrations (see 0.165 and 1.65 µg/mL in Figure 3). The same consideration on the effect of the stiffness of the material on cell viability can be reported for GN2p. When the material became stiffer cells are able to sense the mechanical response of the material surface and interact by modifying their proliferation cues.

### 3.3. Evaluation of Cell–Material Interactions by Imaging of Osteoblast-Like Cells Cultured in the Presence of CS and GN1p Powders

The observation of cell–material interactions on the material surface is regarded as a key determinant for assessing the performance of a biomaterial. Cell morphology, adhesion and spreading provide clear indication about the growth behavior and appropriate cellular responses to the tested materials [36]. Based on the viability results, the morphological cell–material interactions were investigated by culturing the MG63 cells grown in the presence of the CS and GN1p powders. Cell cultures were observed 72 h after exposure to the material powders at different concentrations. The imaging of morphology and adhesion of MG-63 cells proliferating in the presence of (1.5, 0.15 and 0.015 mg/mL) CS or GN1p indicated no evident differences compared to the untreated control cells; in both treatments, cells appeared homogeneously distributed, regularly adhering and migrating on the culture surface, exhibiting their regular thin and elongated shape (data shown in Supplementary Material, Figure S1a,b); neither changes of the morphological phenotype nor inhibition of migration possibly due to the presence of material fragments could be detected compared to the untreated cells (Supplementary Material, Figure S1c,d).

**Figure 4:**
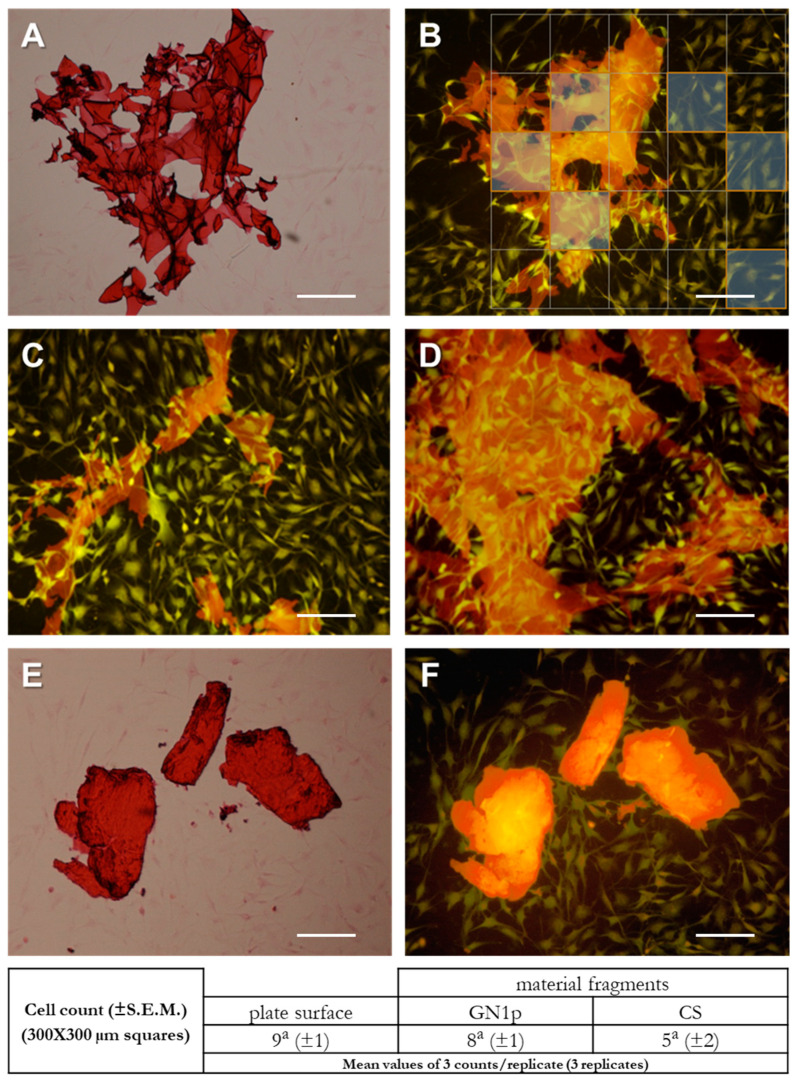
Evaluation of cell–material interactions by imaging of MG63 cells cultured in the presence of CS and GN1p powders. HE staining and fluorescence imaging of MG63 cells grown in the presence of GN1p (**A**–**D**) and CS powder (**E**,**F**). Representative pictures after 72 h growth in the presence of 1.5 mg/mL material powders. (**A**) Bright-field image of the cells growing on a surface area surrounding a GN1p fragment. (**B**) Fluorescence image (red fluorescence) of the same fragment. (**C**,**D**) Additional images showing cell adhesion on GN1p fragments with heterogeneous shapes; pictures show high efficiency of the cell spreading onto the fragments regardless size and morphology. (**E**,**F**) Bright-field and fluorescence images of cells in the presence of a CS powder fragment (stereoscope magnification 10×; scale bar 300 µm; Nikon red fluo filter (mcherry) ex. 562/40 em. 641/75). In B, representative 300 µm × 300 µm squares are reported to depict the selection of the areas for the cell counts. In the table below: cell numbers from three different counts per biological replicate (3 different replicates) were performed; data are reported as mean values (±S.E.M.); statistical analysis: Student’s *t*-test; different/equal letters indicate statistically different/equal values.

A more detailed analysis could be assessed based on the fluorescence imaging of the HE-stained cells. By focusing on the interaction of cells proliferating/migrating in the presence of the material powders, remarkable affinity of MG63 cells for the GN1p material was detected (Figure 4A–D). In fact, cells adhered and migrated onto the surface of the material fragments, i.e., the GN1p surfaces showed to highly favor cell invasiveness within the material’s mesh and infoldings regardless of the 3D structural complexity. Moreover, by fluorescence imaging the absence of morphological differences between cells spreading in the presence or in the absence of GN1p was also confirmed. The cell–CS powder interactions were also analyzed. Interestingly, lower affinity between the spreading cells and the CS material seemed to occur, suggesting a lower permissiveness of the CS fragments in terms of cell adhesion and migration (Figure 4C,D). These evidences were corroborated by bidimensional cell counts performed in selected areas of the culture surface with or without material fragments (see representative Figure 4B). Intriguingly, for GN1p no statistically significant differences were found between the average number of cells counted in areas without fragments (9 ± 1) and the average number of cells counted on the fragments’ surface (8 ± 1; see table in Figure 4); contrariwise, a lower number of cells seemed to be able to migrate and populate the surface of the CS fragments, although without significant difference. It is worth to note that these differences in the behavior of migrating cells and in the material biocompatibility seems to mirror the apparent differences in the surfaces’ roughness as evidenced by the SEM morphological analysis of the material fragments.

### 3.4. Analysis of Pro-/Anti-Inflammatory Responses in THP-1 Monocyte-Like Cells

**Figure 5:**
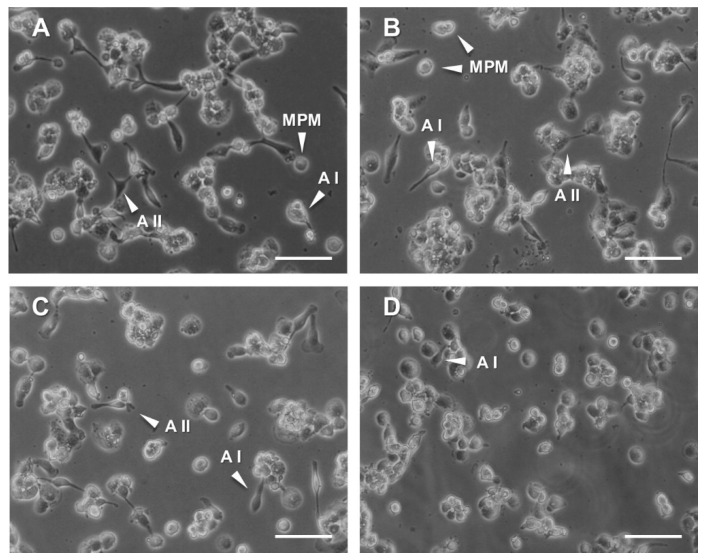
Morphological analysis of THP-1 cells grown in the presence of the material powders or genipin and treated with 100 nM PMA for 48 h. (**A**) Positive control of THP-1 cells treated by the only PMA inflammatory stimulus. (**B**) PMA-treated cells + CS powder (0.15 mg/mL). (**C**) PMA-treated cells + GN1p (0.15 mg/mL); the representative picture is comparable to data not shown for the GNp and GN2p treatments. (**D**) PMA-treated cells + gn (0.022 µg/mL). MPM: monocytes pre-macrophage cells; AI: adhered stage I, cells showing low morphological complexity, with *n* = 1 pseudopodial elongation; AII: adhered stage II, adhered cells with *n* ≥ 2 pseudopodial elongations and advanced morphological complexity (Bright field images, 10× magnification, scale bar: 50 µm).

The evaluation of possible anti-inflammatory properties of the material powders has been assessed in the human-derived THP-1 cells exposed to the PMA-mediated inflammatory stimulus. Cells grown in the presence of 0.15 mg/mL CS powder, 0.15 mg/mL GNp, GN1p and GN2p or the corresponding gn content (0.022, 0.075 and 0.165 µg/mL, respectively) were treated with PMA for 48 h. Cells undergoing the only PMA inflammatory stimulus were used as a positive control. THP-1 have been largely used to monitor significant changes in cell morphology/morphometry related to the monocyte–macrophage transition consequent to chemical, physical and/or mechanical stimuli [37,38,39,40,41], in addition to targeted molecular assays for evaluating the differentiation of THP1 cell to monocyte-derived macrophages, e.g., by identifying surface markers and production of certain cytokines [42]. We analyzed the THP-1 morphology changes as early hallmarks of the macrophagic induction. Being the pro-inflammatory PMA treatments able to elicit the suspension–adhesion transition of cells, their morphological spreading and formation of pseudopodia and elongations [42], we evaluated the basic effects of PMA on THP-1 adhered monocytes in terms of their initial progression of morphological complexity. In this view, we focused on three stages/shapes: (a) adhered monocyte-like cells (MPM; monocytes, pre-macrophage); (b) adhered spindle-shaped cells with single pseudopodial elongation and low morphological complexity (AI; adhered stage I) and (c) adhered cells with *n* ≥ 2 pseudopodial elongations and advanced morphological complexity (AII; adhered stage II). By analyzing the morphology of cells adhered to the culture plates, differences could be detected among the positive control (i.e., cells treated with PMA alone) and all the material/genipin treatments. As expected, the PMA-treated control cells underwent transition, adhering to the culture plate and showing diverse shapes and heterogeneous morphological complexity, from globular (MPM) to elongated, and star shapes with mono- (A I) or multiple (A II) pseudopodial formations (Figure 5A). Interestingly, cells grown in the presence of CS powder and treated with PMA adhered to the culture plate showing the same heterogeneous morphological pattern of the PMA-treated positive control (Figure 5B). Even more interesting were the observations on THP-1 cells treated with GNp, GN1p and GN2p material powders and stimulated by PMA (Figure 5C); in fact, adhered cells predominantly revealed a rounded shape (MPM) rather than elongated, with less presence of star-shaped cells. Finally, PMA-treated cells, which were exposed to 0.022 µg/mL free gn (i.e., corresponding to the GNp genipin content), showed morphology and adhesion patterns similar to those of the GNp, GN1p and GN2p conditions with the apparent increase of the MPM rounded cells (Figure 5D). It has to be noticed that in our hands the PMA-treated cells exposed to higher gn concentrations (i.e., 0.075 and 0.165 µg/mL corresponding to GN1p and GN2p) mainly showed very limited adhesion; moreover, only MPM adhered cells could be detected, while A I and A II cells were almost absent (data not shown). In this respect, besides the limited adhesion and monocyte–macrophage transition of THP-1 cells, such higher concentrations of free gn may induce complex responses implying the triggering of unresponsive and/or quiescent states, which specifically deserve further investigation.

**Figure 6:**
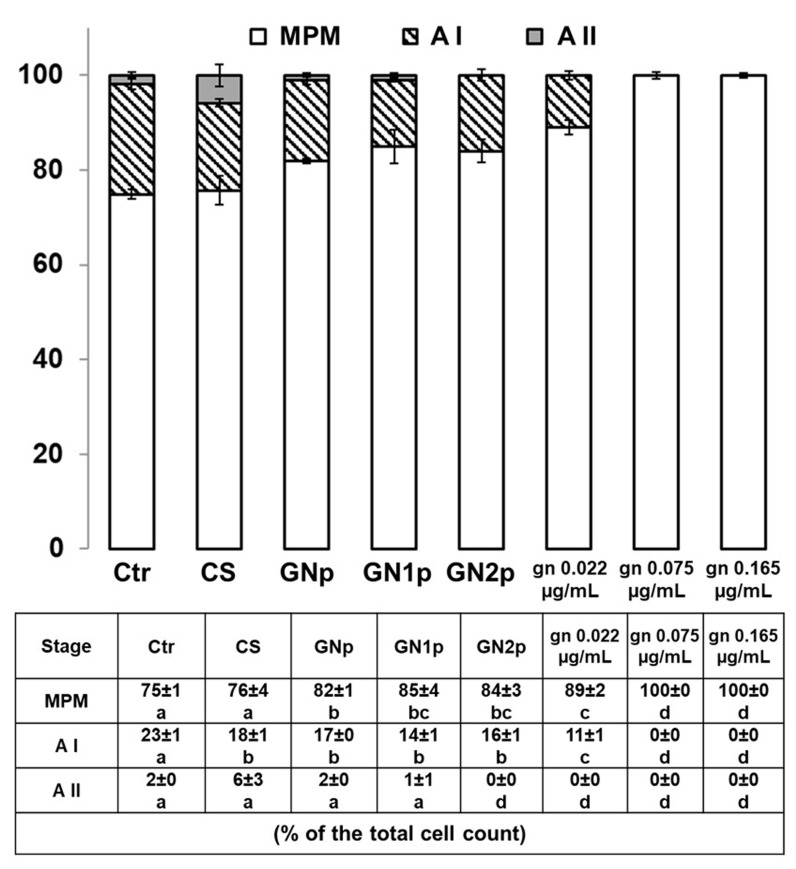
Plotting of the cell counts for the MPM, A I and A II stages. For each treatment condition, the table below reports each percentage (±S.E.M.) with respect to the total cell count (100%). Cell counts were performed in 4 different areas of the culture plate, of 2 biological replicates. Statistical analysis: Student’s *t*-test; different/equal letters indicate statistically different/equal values (*p* < 0.05). MPM: monocytes pre-macrophage cells; AI: adhered stage I, cells showing low morphological complexity, with *n* = 1 pseudopodial elongation; AII: adhered stage II, adhered cells with *n* ≥ 2 pseudopodial elongations and advanced morphological complexity.

Based on the qualitative observations, counting of cells of the MPM, A I and A II stages were performed for a relative-quantitative assessment of the THP-1 cellular responses to the pro-inflammatory stimulus of PMA. As summarized in Figure 6, the count results overall confirmed the qualitative observations. First, the numbers of adhered monocyte-like cells (MPM) as well as of A I and A II cells (low and advanced morphological complexity) was comparable between the PMA-treated positive control and the PMA/CS-treated cells; in particular, in the presence of CS an increasing of the number of cells at the A II stage seems to occur. Remarkably, when PMA-treated cells are exposed to the GNp, GN1p and GN2p material powders, the number of MPM cells is increased with respect to CS while the number of A I and A II cells is decreased: noteworthy, the MPM increase and the A I-II decrease are parallel to the increasing amount of genipin in the GNp, GN1p and GN2p materials. In other words, as the plotted data of cell counts suggest (Figure 6), CS seems to induce the A II stage more than the genipin-containing powders, which in turn hold higher potential to avoid the monocyte–macrophage transition. These trends are coherently enhanced by the analyses of the effects of PMA on cells exposed to the free gn solutions. In fact, the number of cells at the MPM stage was found to increase further. Cell counting analyses showed that CS crosslinked by genipin generated a cell response that was in between the positive control (PMA-treated cells) and the PMA-gn-treated cells. Additionally, these data suggest that genipin can be used as a crosslinking agent for the CS biomaterial while limiting the differentiation of monocytes into macrophages (Figure 6).

## 4. Conclusions

The evaluation of the basic biocompatibility of CS, together with the disclosure of potential anti-inflammatory properties of genipin, shows the potential in using genipin biomaterials as bioresorbable implants. Overall, our experiments with the osteoblast-like cells showed that specific combination of CS and genipin (e.g., the GN1p material) promotes the proliferation and migration of cells, which also implies tissue integration properties higher than the pure CS. On the other hand, the evidence from the monocyte-like cells indicated that the use of genipin as a cross-linker seems to reduce the reported CS-induced monocyte–macrophage transition, at least at the morphological level. These properties can be exploited to enhance the modularity of pro-inflammatory versus anti-inflammatory behavior of the CS-based biomaterials to promote bone regeneration by a tissue engineering approach. By exposing both the studied cell lines to materials powders, the study was able to provide new insights on the cell–material interactions occurring during the process of resorption of CS scaffolds. Further investigations are needed to reveal in detail the impact on gene expression that may be causing the observed differences in biocompatibility. To this end, the analysis of marker proteins possibly belonging to the adeshome-inflammasome intersection network may represent, intriguingly, the basis, currently lacking, for the experimental steps necessary to move towards the in vivo validation of the investigated materials.

Nevertheless, in this work, the role of genipin as crosslinker of CS has been found to have the additional advantage of improving the biomaterial biocompatibility. Moreover, results could suggest the opportunity to use these materials not only for tissue engineering applications, but also for targeted therapeutic treatments of inflammed injuries. Genipin therefore emerges as a key element for the success of CS as a biomaterial. Unlike other crosslinker, genipin is non-toxic. On the other hand, it is a bioactive molecule exerting a key activity on cells involved in the tissue repair process. This is a step forward towards the establishment of pharmacological effects that can be exploited in bioactive scaffolding systems able to finely tune the role of inflammatory cells towards a regenerative phenotype while avoiding the chronic inflammation and the resulting fibrotic capsule.

## Supplementary Materials

The following are available online at https://www.mdpi.com/2079-7737/9/7/159/s1, Supplementary material S1, Figure S1: Qualitative evaluation of cell morphology and proliferation on the growth surface as assessed by HE staining of MG63 cells grown in the presence of CS powder (a) and GN1p (b).
