# Supplementary material for: Assessment of Cytocompatibility and Anti-Inflammatory (Inter)Actions of Genipin-Crosslinked Chitosan Powders
Source: Biology (Basel). 2020 Jul 8;9(7):159. doi: 10.3390/biology9070159 (PMC7407416; doi:10.3390/biology9070159)
Supplement: Supplementary file 1 [file biology-09-00159-s001.pdf]

Assessment of cytocompatibility and anti-inflammatory (inter)actions of genipin-crosslinked chitosan powders

Dimida S[1], Santin M[2], Verri T[3], Barca A[3*§], Demitri C[1*§]

[§]Corresponding authors. *Contributed equally to the work.

[1] Biomaterial Laboratory, Department of Innovation for Engineering, University of Salento c/o Ecotekne, 73100 Lecce, Italy

[2] Centre for Regenerative Medicine and Devices, School of Pharmacy and Biomolecular Sciences, University of Brighton, Brighton, United Kingdom

[3]Applied Physiology Laboratory, Department of Biological and Environmental Sciences and Technologies (DeBEST) University of Salento c/o Ecotekne, 73100 Lecce, Italy

Supplementary material S1

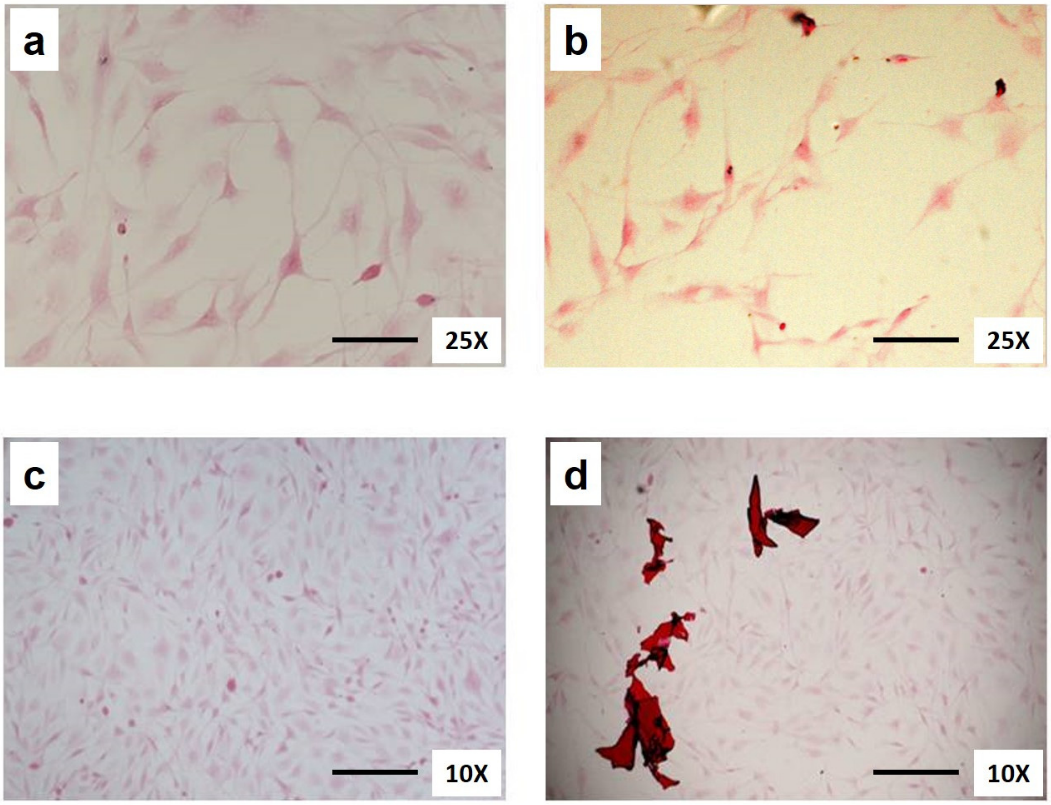

Figure. S1. Qualitative evaluation of cell morphology and proliferation on the growth surface as assessed by HE staining of MG63 cells grown in the presence of CS powder (a) and GN1p (b). In both sample types, cells appeared homogeneously distributed, regularly adhering and migrating on the culture surface, exhibiting their regular thin and elongated shape. Compared to untreated control cells (c), neither changes of the morphological phenotype nor inhibition of migration/proliferation possibly due to the presence of material fragments could be detected (d). Pictures are representative of cells after 72 h treatment with 1.5 mg/ml material powders [Magnif. 25X in a,b and 10X in c,d. Scale bar: 100 μm in a,b and 250 μm in c,d].
